# Supplementary material for: Seq2Bind webserver for binding site prediction from sequences using fine-tuned protein language models
Source: NAR Genom Bioinform. 2025 Nov 22;7(4):lqaf154. doi: 10.1093/nargab/lqaf154 (PMC12639246; doi:10.1093/nargab/lqaf154)
Supplement: lqaf154_Supplemental_Files [file lqaf154_supplemental_files.zip › Supplementary File copy_clean.docx]

Supplementary Figure 1: Distribution of success rate by different patch sizes (1,5,10) and models.


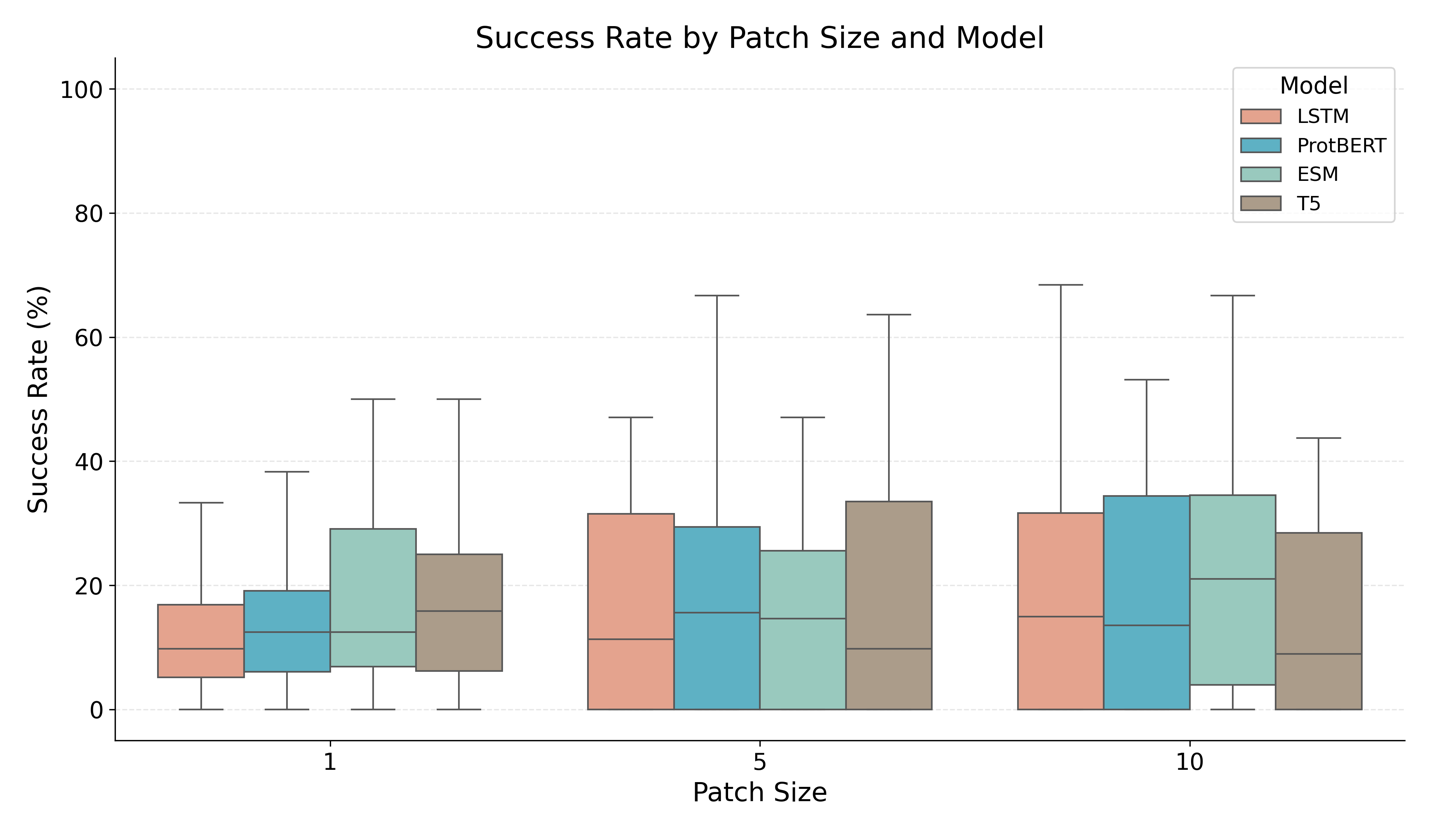


Supplementary Figure 2: LSTM model performance for 6T36 complex (chain A and B) across different N factors.


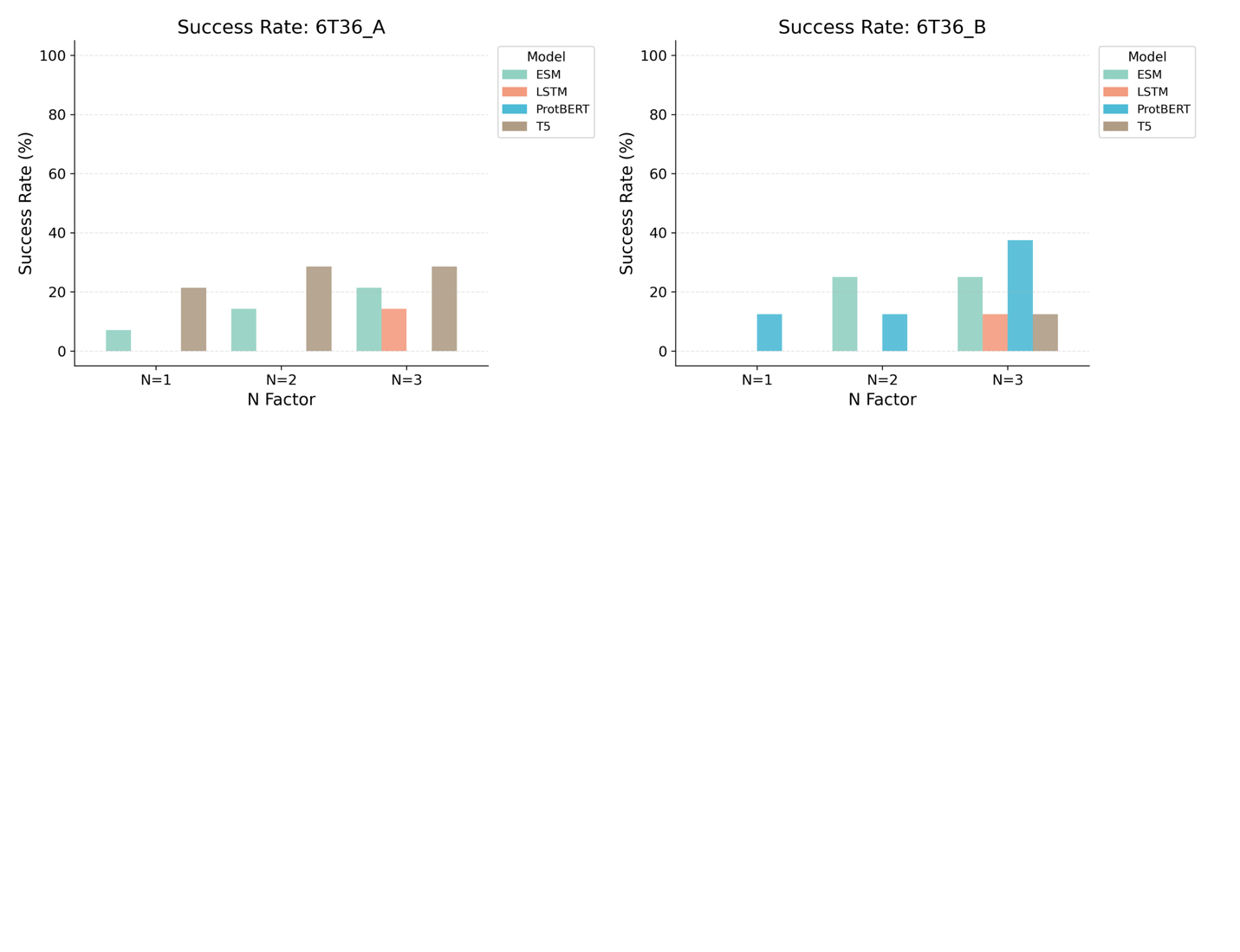


Supplementary Figure 3: Performance of different models across all 14 PDB complexes.


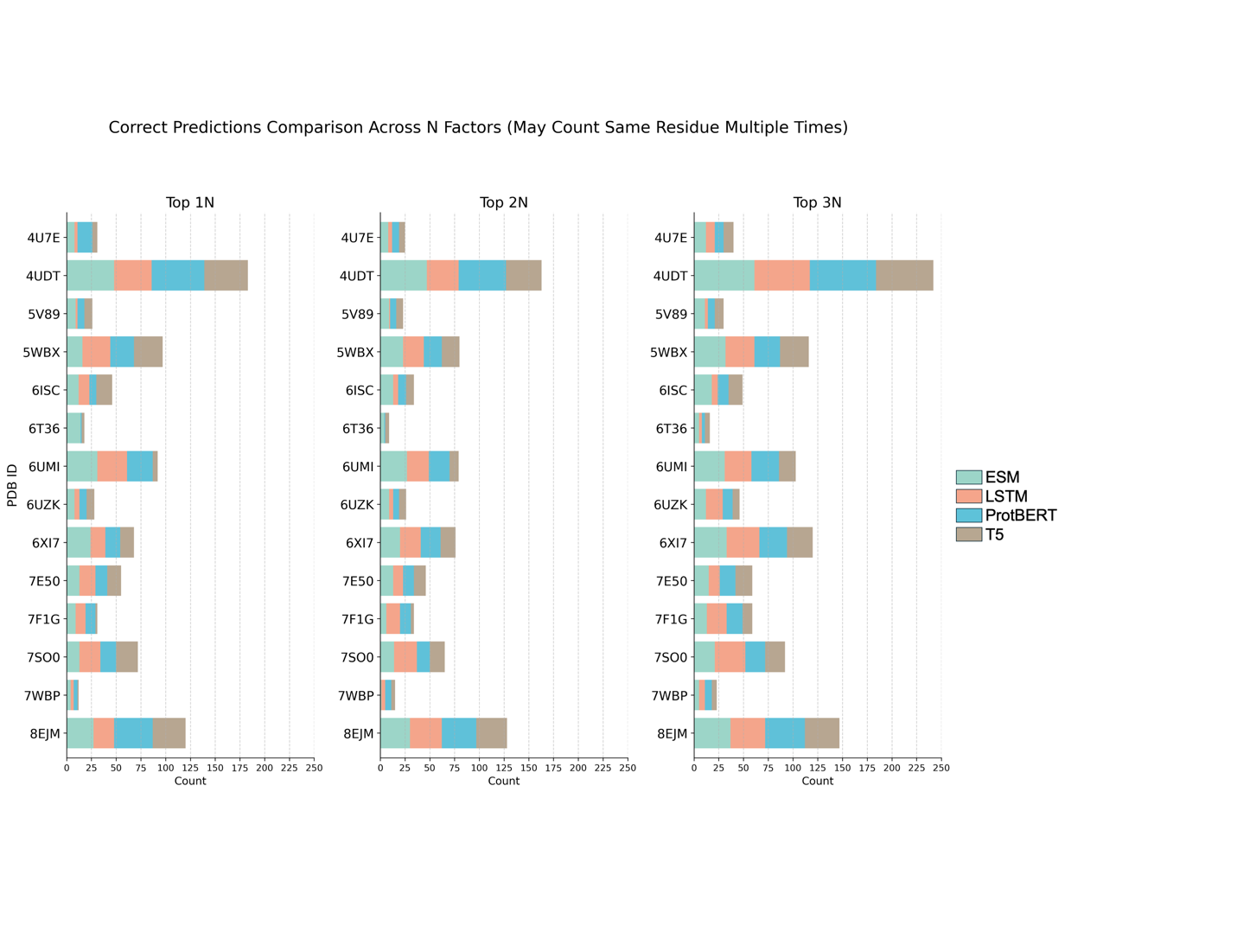


Supplementary Figure 4: Distribution of correctly predicted interaction types across 14 protein complexes, shown as the number of ground truth interactions recovered by each method.


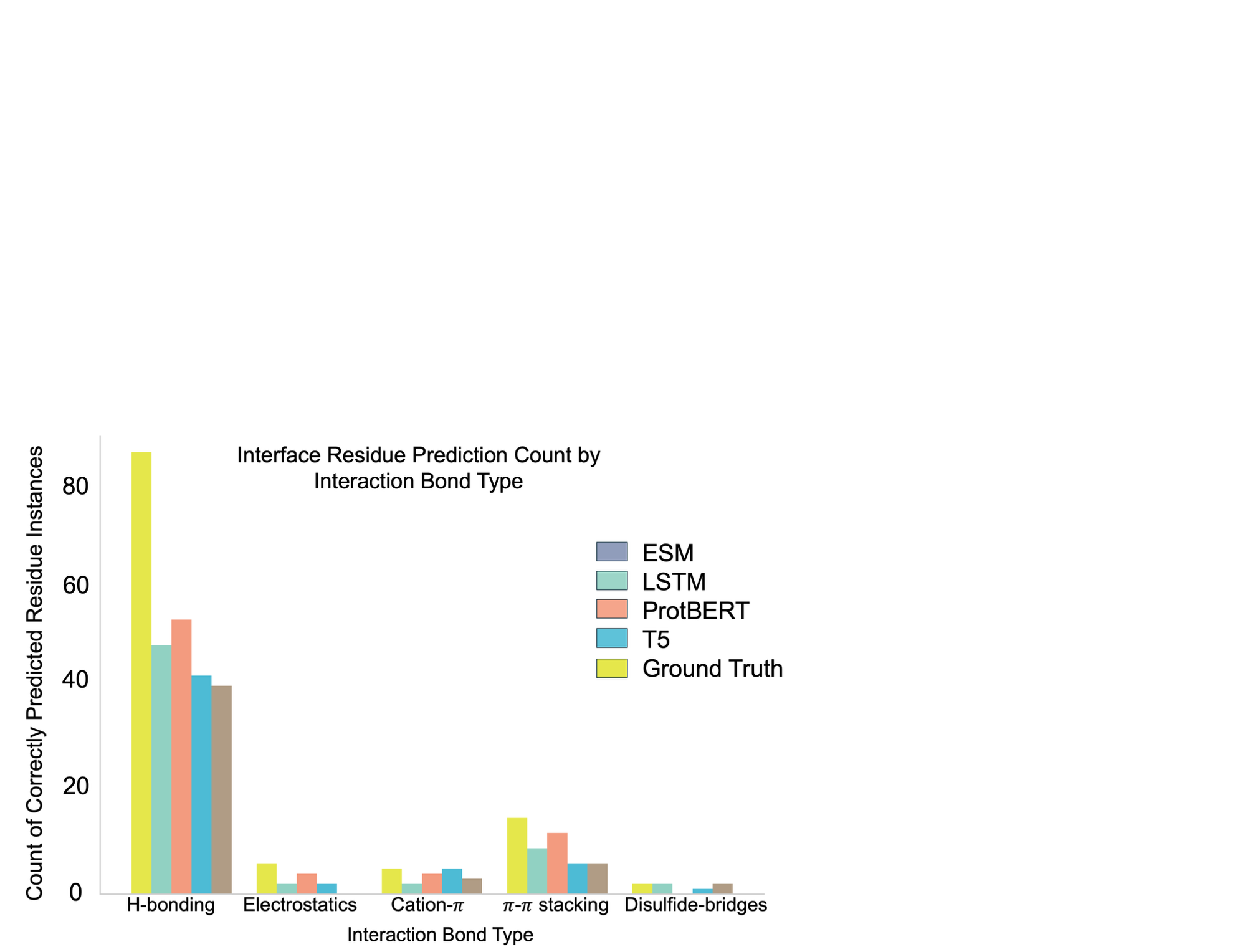


Supplementary Figure 5: Gain and loss of success rate across 4 models across various patch sizes.


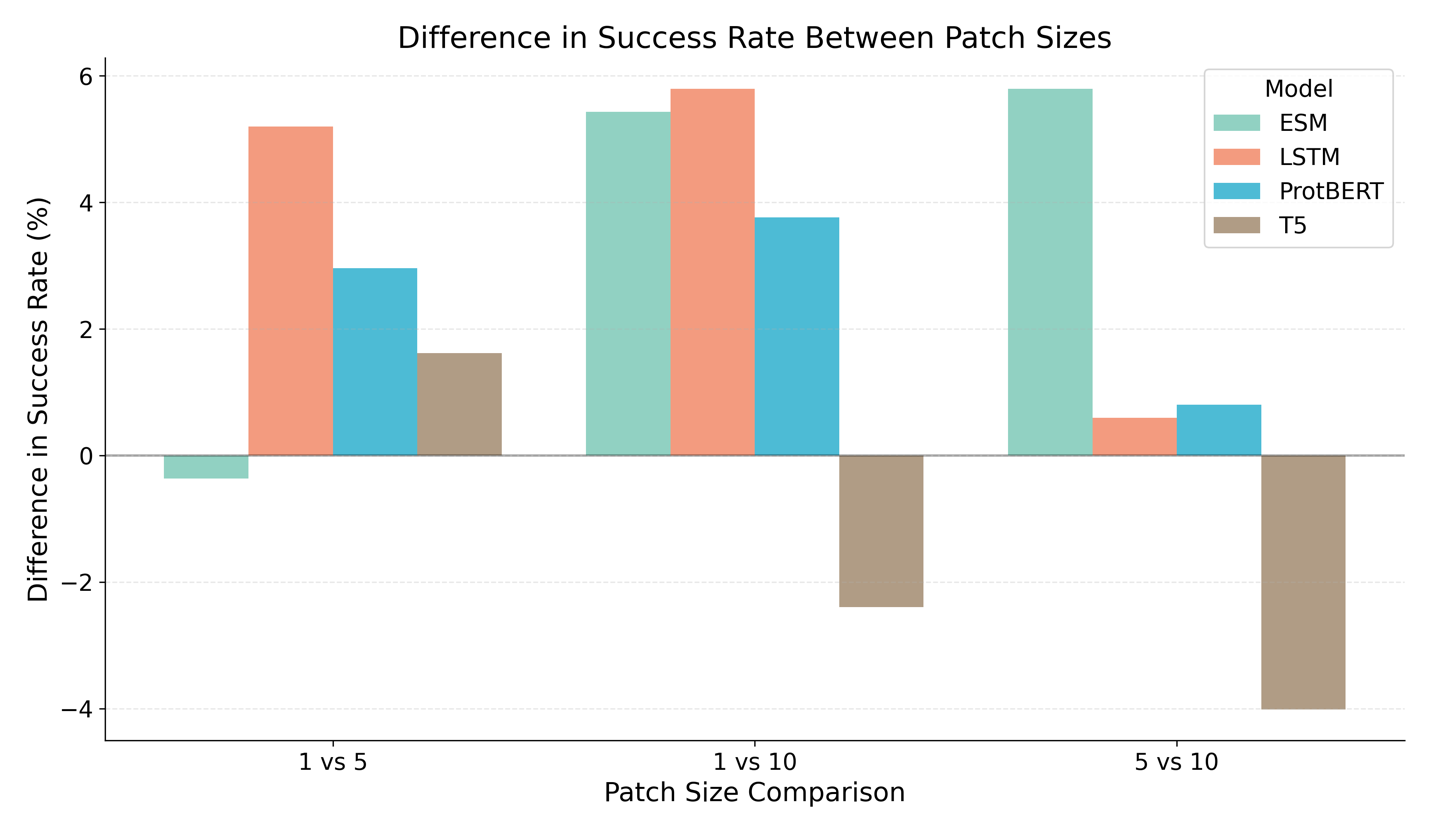


Supplementary Figure 6: Stabilizing vs destabilizing outcome predictions by ESM, BiLSTM, ProtBERT, and T5.


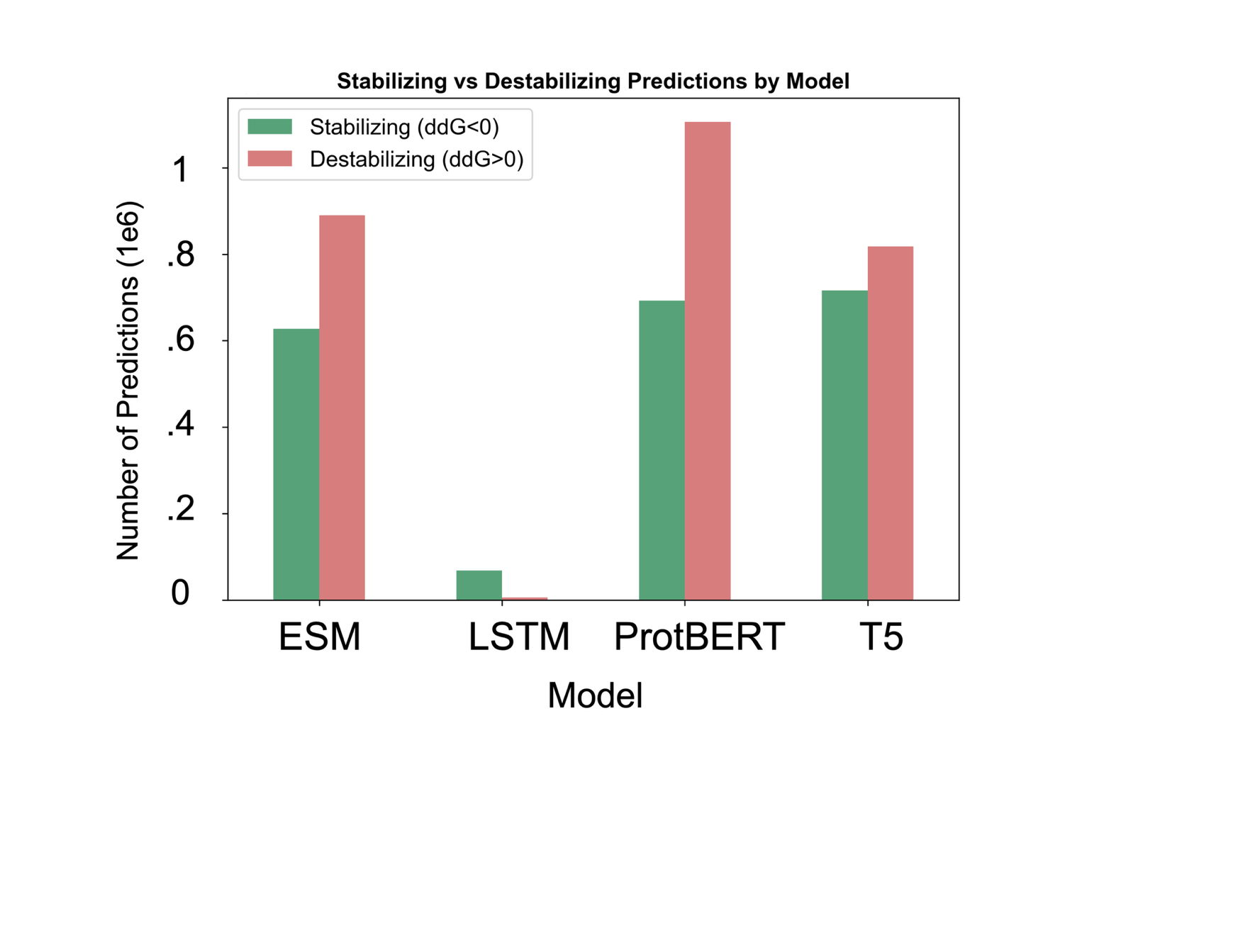


Supplementary Figure 7: Success rate comparison by N factors up to 5.


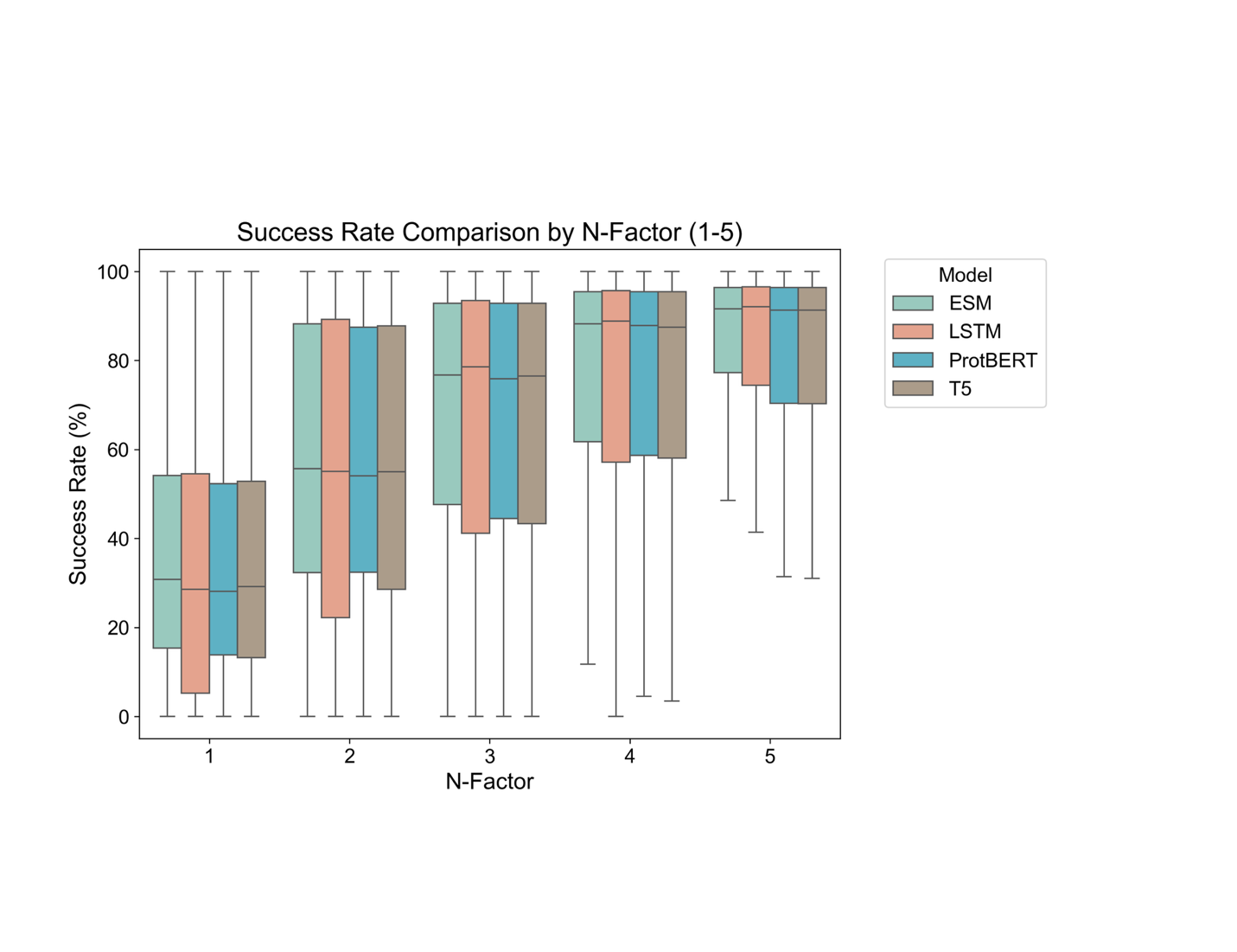


Supplementary Figure 8: An overview of 6,063 protein complex’s length and actual interacting residue numbers.


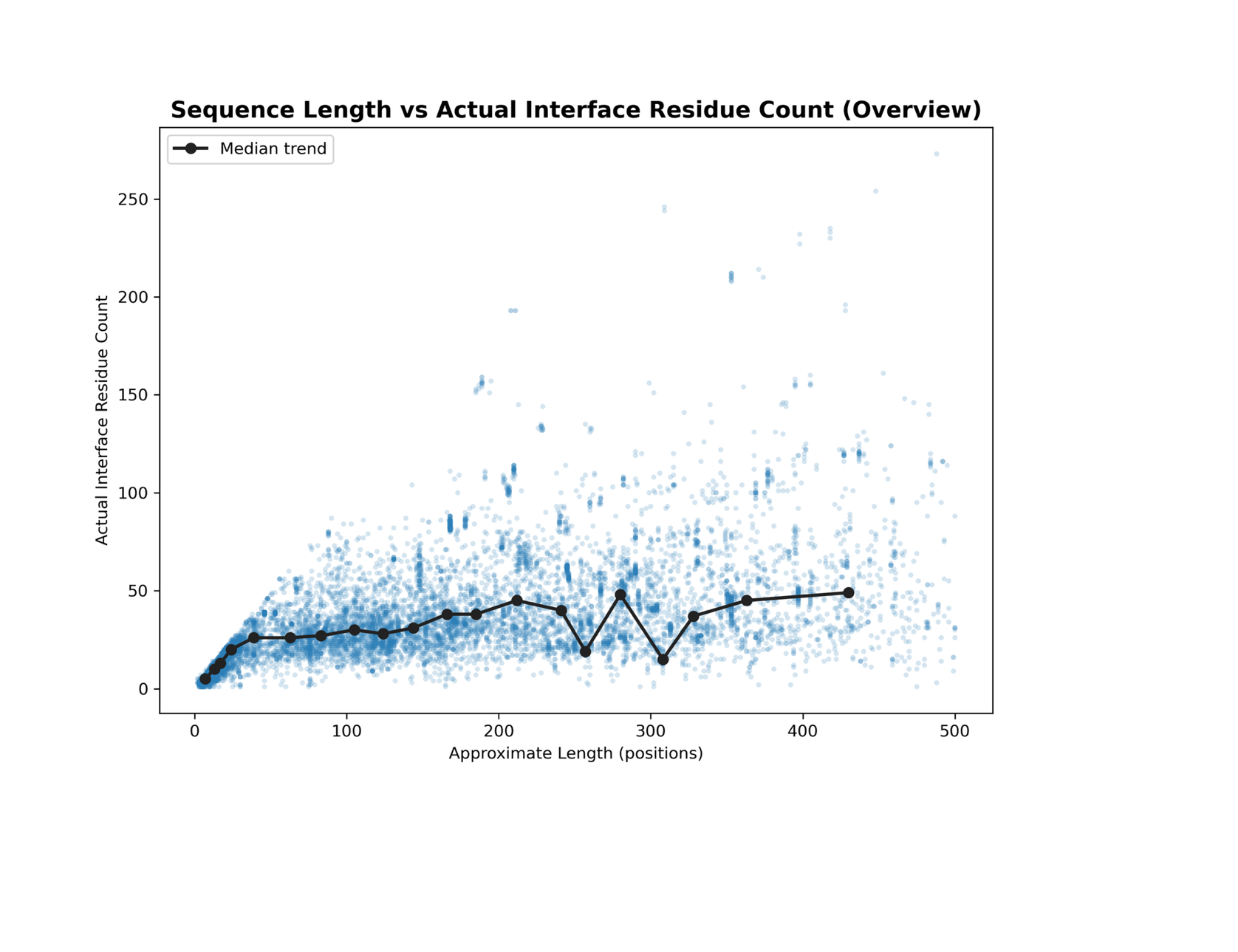


Supplementary Figure 9: Violin plot showing distance distribution and contact type exhibited by the predicted residues with the closest residue in the true interfacial region.


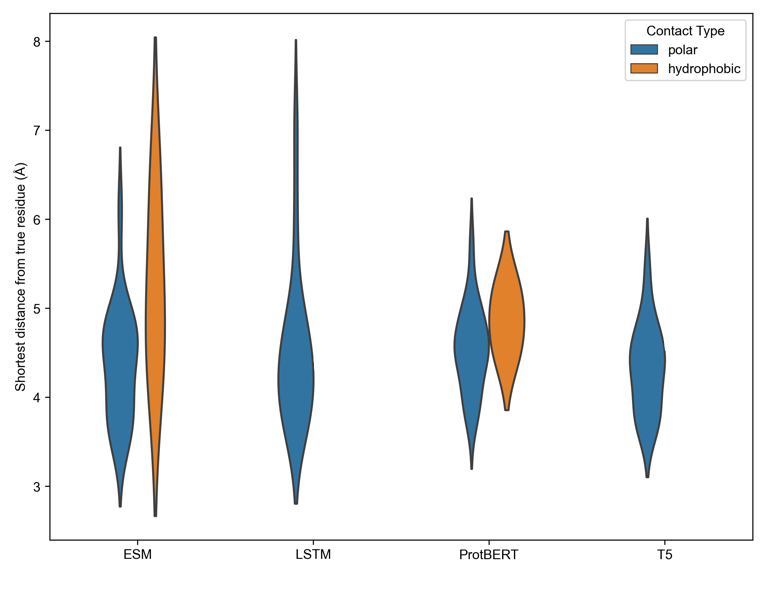


Supplementary Table 1: An overview of the 14 selected PDBs for comparison with other SOTA methods with Seq2Bind and their significance.

| **PDB** | **Complex (partners)** | **Why it matters** |
| --- | --- | --- |
| **4U7E** | LIP5 (VTA1) N-terminal domain ↔ IST1 MIT-interacting motif (ESCRT-III) | Defines MIT–MIM recognition in ESCRT, a membrane-remodeling machine essential for cytokinesis, MVB biogenesis and virus budding. (1) |
| **4UDT** | Staphylococcal enterotoxin E (SEE) superantigen ↔ human T-cell receptor (TRBV7-9) | Structural basis of superantigen-driven T-cell activation/toxic shock, an immunopathology benchmark complex. (2) |
| **5V89** | Human DCN1-like protein 4 (DCN4) PONY domain ↔ CUL1 WHB domain | Captures an N-terminal acetylation-dependent E3-ligase interaction needed for cullin neddylation; small-molecule disruption blocks ligase activity (drug discovery relevance). (3) |
| **5WBX** | Calmodulin ↔ SK/IK (KCNN) channel calmodulin-binding domain + positive modulators | Explains how small molecules potentiate SK/IK K⁺ channels that shape neuronal/cardiac excitability—widely used in channel pharmacology. (4) |
| **6ISC** | CD226 (DNAM-1) ectodomain ↔ CD155 (Necl-5) | Defines a key NK/T-cell immune-checkpoint interaction with implications for anti-tumor immunity. (5) |
| **6T36** | Human PTPN3 PDZ domain ↔ Hepatitis B virus core C-terminal peptide | Viral–host PDZ interaction that may modulate HBV biology; provides an antiviral targetable interface. (6) |
| **6UMI** | CGRP receptor ↔ erenumab (therapeutic mAb Fab) | Mechanism of migraine prevention by antibody blockade of CGRP-R; explains clinical selectivity. (7) |
| **6UZK** | KRIT1 FERM ↔ HEG1 tail (± inhibitor HKi-6) | Structural basis to inhibit HEG1–KRIT1; pharmacologic disruption elevates KLF2/KLF4 in endothelium (vascular biology). (8) |
| **6XI7** | KRAS-GTPase ↔ RAF1 RBD + CRD | Near-full RAS–RAF activation interface; central to oncogenic MAPK signaling and inhibitor design. (9) |
| **7E50** | Microplasmin (trypsin-like protease) ↔ Aedes aegypti Kazal inhibitor (AaTI) | Serine-protease inhibition blueprint with links to fibrinolysis/antithrombotic strategies. (10) |
| **7F1G** | Human BACE2 ↔ selective small-molecule inhibitor | BACE1/BACE2 selectivity insights for Alzheimer’s-related amyloid processing; medicinal-chemistry benchmark. (11) |
| **7SO0** | Engineered tick evasin **EVA-P974(F31A)** ↔ human chemokine **CCL2 (MCP-1)** | Structure reveals how engineered evasins bind and neutralize pro-inflammatory CC chemokines, offering a blueprint for anti-inflammatory therapeutics. (12) |
| **7WBP** | SARS-CoV-2 Omicron spike RBD ↔ human ACE2 | Variant-specific receptor engagement underlying transmissibility/immune escape. (13) |
| **8EJM** | Human DHX15 helicase ↔ SUGP1 G-patch | Explains DHX15–SUGP1 coupling in splicing, which links to SF3B1-mutant mis-splicing in cancer. (14) |

References

1. Guo,E.Z. and Xu,Z. (2015) Distinct mechanisms of recognizing endosomal sorting complex required for transport III (ESCRT-III) protein IST1 by different microtubule interacting and trafficking (MIT) domains. *Journal of Biological Chemistry*, 290, 8396–8408.

2. Rödström,K.E.J., Regenthal,P. and Lindkvist-Petersson,K. (2015) Structure of staphylococcal enterotoxin E in complex with TCR defines the role of TCR loop positioning in superantigen recognition. *PLoS One*, 10, e0131988.

3. Scott,D.C., Hammill,J.T., Min,J., Rhee,D.Y., Connelly,M., Sviderskiy,V.O., Bhasin,D., Chen,Y., Ong,S.-S. and Chai,S.C. (2017) Blocking an N-terminal acetylation–dependent protein interaction inhibits an E3 ligase. *Nat Chem Biol*, 13, 850–857.

4. Nam,Y.-W., Orfali,R., Liu,T., Yu,K., Cui,M., Wulff,H. and Zhang,M. (2017) Structural insights into the potency of SK channel positive modulators. *Sci Rep*, 7, 17178.

5. Wang,H., Qi,J., Zhang,S., Li,Y., Tan,S. and Gao,G.F. (2019) Binding mode of the side-by-side two-IgV molecule CD226/DNAM-1 to its ligand CD155/Necl-5. *Proceedings of the National Academy of Sciences*, 116, 988–996.

6. Genera,M., Quioc-Salomon,B., Nourisson,A., Colcombet-Cazenave,B., Haouz,A., Mechaly,A., Matondo,M., Duchateau,M., König,A. and Windisch,M.P. (2021) Molecular basis of the interaction of the human tyrosine phosphatase PTPN3 with the hepatitis B virus core protein. *Sci Rep*, 11, 944.

7. Garces,F., Mohr,C., Zhang,L.I., Huang,C.-S., Chen,Q., King,C., Xu,C. and Wang,Z. (2020) Molecular insight into recognition of the CGRPR complex by migraine prevention therapy Aimovig (Erenumab). *Cell Rep*, 30, 1714-1723. e6.

8. Lopez‐Ramirez,M.A., McCurdy,S., Li,W., Haynes,M.K., Hale,P., Francisco,K., Oukoloff,K., Bautista,M., Choi,C.H.J. and Sun,H. (2021) Inhibition of the HEG1–KRIT1 interaction increases KLF4 and KLF2 expression in endothelial cells. *FASEB Bioadv*, 3, 334–355.

9. Tran,T.H., Chan,A.H., Young,L.C., Bindu,L., Neale,C., Messing,S., Dharmaiah,S., Taylor,T., Denson,J.-P. and Esposito,D. (2021) KRAS interaction with RAF1 RAS-binding domain and cysteine-rich domain provides insights into RAS-mediated RAF activation. *Nat Commun*, 12, 1176.

10. Walvekar,V.A., Ramesh,K., Jobichen,C., Kannan,M., Sivaraman,J., Kini,R.M. and Mok,Y.K. (2022) Crystal structure of Aedes aegypti trypsin inhibitor in complex with μ‐plasmin reveals role for scaffold stability in Kazal‐type serine protease inhibitor. *Protein Science*, 31, 470–484.

11. Ueno,T., Matsuoka,E., Asada,N., Yamamoto,S., Kanegawa,N., Ito,M., Ito,H., Moechars,D., Rombouts,F.J.R. and Gijsen,H.J.M. (2021) Discovery of extremely selective fused pyridine-derived β-site amyloid precursor protein-cleaving enzyme (BACE1) inhibitors with high in vivo efficacy through 10s loop interactions. *J Med Chem*, 64, 14165–14174.

12. Bhusal,R.P., Aryal,P., Devkota,S.R., Pokhrel,R., Gunzburg,M.J., Foster,S.R., Lim,H.D., Payne,R.J., Wilce,M.C.J. and Stone,M.J. (2022) Structure-guided engineering of tick evasins for targeting chemokines in inflammatory diseases. *Proceedings of the National Academy of Sciences*, 119, e2122105119.

13. Han,P., Li,L., Liu,S., Wang,Q., Zhang,D., Xu,Z., Han,P., Li,X., Peng,Q. and Su,C. (2022) Receptor binding and complex structures of human ACE2 to spike RBD from omicron and delta SARS-CoV-2. *Cell*, 185, 630-640. e10.

14. Zhang,J., Huang,J., Xu,K., Xing,P., Huang,Y., Liu,Z., Tong,L. and Manley,J.L. (2022) DHX15 is involved in SUGP1-mediated RNA missplicing by mutant SF3B1 in cancer. *Proceedings of the National Academy of Sciences*, 119, e2216712119.
